# 

**Published:** 1849-07

**Authors:** 


### NOTICE TO READERS AND CORREFPONDENTS.

We have received original communications from Drs. Thomas Hall, T. Bullard, E. S Cooper, J. J. Lesher, Tildon, E. C. Banks, Proceedings of the Annual meeting of the Rock River Medical Socie,y. Trial for mal-practice from Dr. Spencer. Proceedings of the Wisconsin State Medical Society.

We have received, too late for notice, in this number, the following Works, which will be attended to in our next. From Lea & Blanchard, Smith on Parturition, for sale by J. Keen, jr. & Brother. Kirks & Paget's Physiology. Bowman's Practical Chemistry.—Lee's Clinical Midwifery. Day on Diseases of Old Age; for sale by Griggs, Bross & Co.

From Lindsay & Blakiston, Noad's Chemical Analysis, by Morfit. Bull on the Maternal management of Children; for sale by Griggs, Bross & Co. From Tickner & Co., Channing on Etherization in Midwifery; Warren on Etherization; Warren on Chloroform and Chloric Ether, and Warren on the Preservation of Health.

The following pamphlets have been received:—Speech in support of the memorial of Harvard, Williams and Amherst Colleges, by Hon. Edward Everett. Report of the Trustees and Superintendent of the Butler Hospital for the Insane. Report of the Trustees of the Massachusetts General Hospital. Second Annual Report of the Trustees of the Indiana Institute for the Blind. Mr. Cushing's Starling Medical College Address. Dr. Ramsay's Letters upon Cholera. Proceedings of the Medieal Convention in Ohio. Prof. White's remarks upon Obstetrical Forceps. Catalogue and Circular of the Medical Department of the Univ. of Missouri. Prof. Gibbon's Introductory Lecture to the Summer Course in the Philadelpeia College of medicine. Catalogue of Books for sale by Samuel S. & William Wood, New York. Catalogue of Books for sale by Tickner & Co., Boston

☞ The crowded condition of our pages, precludes the possbility of inserting more than a mere acknowledgement of the receipt of the above named pamphlets. Some of them we will endeavor to notice more fully hereafter.

---

### RECEIPTS FOR THIS JOURNAL FOR MAY AND JUNE, 1849.

| | |
|---|---|
| **INDIANA.** | |
| Drs. L. Wallace, | $2 00 |
| J. Hendricks, | 2 00 |
| J. Courtney, | 2 00 |
| Luther Jewett, | 5 00 |
| J. H. Dunlap, | 2 00 |
| J. G. Osborne, | 4 00 |
| Arthur Bigger, | 2 00 |
| P. Q. Stryker, | 4 00 |
| S. B. Bushnell, | 4 00 |
| W. Anderson, | 5 00 |
| J. H. Constant, | 2 00 |
| James Ely, | 2 00 |
| C. Brown, | 1 50 |
| G. M. Huggins, | 1 50 |
| A. W. Armstrong, | 1 50 |
| L. Leyman, | 4 0[illegible] |
| E. Allen, | 2 00 |
| Z. Doherty, | 2 00 |
| John McCorkle, | 2 00 |
| Jefferson Miller, | 2 00 |
| G. D. Becker, | 1 00 |
| W. J. Walker, | 2 00 |
| J. Holman, | 2 00 |
| H. Duncan, | 4 00 |
| A. S. Ward, | 4 00 |
| R. C. Bond, | 2 00 |
| W. Dickey, | 2 00 |
| J. E. Moler, | 2 00 |
| J. P. Tucker, | 4 00 |
| Davis & Wigans, | 4 00 |
| Leonard Barber, | 2 00 |
| R. H. Tarleton, | 2 00 |
| M. W. Thomas, | 2 00 |
| D. D. Hall, | 2 00 |
| Jas. Kinkennon, | 2 00 |
| U. A. V. Hester, | 2 00 |
| T. B. Thompson, | 3 00 |
| J. M. Wells, | 2 00 |
| Ind. Hosp., for Insane, | 4 00 |
| M. Robbins, | 2 00 |
| A. V. Talbott, | 3 00 |
| U. Farquhar, | 4 00 |
| J. S. Cole, | 2 00 |
| R. Brown, | 3 00 |
| Drs R C Moore, | 2 00 |
| D B H Skinner, | 2 00 |
| A Poteet, | 2 00 |
| J P. Dimmitt, | 2 00 |
| D Hutchinson, | 4 00 |
| L A Johnson, | 2 00 |
| S P Mendenhall, | 1 00 |
| J T Pratt, | 2 00 |
| W Brenton, | 4 00 |
| J H Harryman, | 2 00 |
| Mahan & Crowder, | 2 00 |
| Allen and Weaver, | 4 00 |
| J. Depew, | 4 00 |
| J. Stelson, | 0 32 |
| B. Newton, | 1 00 |
| R. C. Hamill, | 2 00 |
| Dr. Smydth, | 2 00 |
| J. H. Wakefield, | 2 00 |
| Buoyer, | 4 00 |
| A. Holmes, | 2 00 |
| W. G. Wood, | 4 00 |
| A. J. Minick, | 4 00 |
| J. M. Stucky, | 4 00 |
| E. Johnson, | 2 00 |
| **ILLINOS.** | |
| Drs. J. Pearson, | 2 00 |
| S. Wiley, | 2 00 |
| J. G. Tilden, | 4 00 |
| Joseph Teft, | 2 00 |
| S. Christy, | 4 00 |
| G. H. Hickman, | 2 00 |
| Chas. Hansford, | 2 00 |
| Samuel Thompson, | 2 00 |
| P. Dickinson, | 4 00 |
| N. D. Sweetland, | 4 00 |
| H. Todd, | 1 00 |
| S. F. Avery, | 2 00 |
| J. S. Whitmire, | 2 00 |
| H. W. Cass, | 1 00 |
| Samuel Elder, | 3 00 |
| D. Wheeler, | 2 00 |
| Silas Meacham, | 2 00 |
| C. C. Bradley, | 4 00 |
| G. W. Southwick, | 2 00 |
| Wm. Brooks, | 2 00 |
| S Vreedenburg, | 3 00 |
| Alvin Ballow, | 3 00 |

| | |
|---|---|
| L. W. Hess, | 2 00 |
| M. T. Steel, | 2 00 |
| J. M. Logan, | 2 00 |
| E. S. Patton, | 2 00 |
| T. Stewart. | 2 00 |
| Chas. Johnson, | 2 00 |
| Richard McGee, | 2 00 |
| J. W. Freer, | 2 00 |
| A. Smith, | 2 00 |
| D. M. Camerer, | 4 00 |
| James Adair, | 2 00 |
| L. Q. Newton, | 4 00 |
| E. Smith, | 3 00 |
| J. H. Syford, | 4 00 |
| H L. Merriam, | 2 00 |
| C. H. Richings, | 2 00 |
| Wesly Pierce, | 4 00 |
| Thos. C. Doge, | 2 00 |
| A. G. Porter, | 1 00 |
| M. D. Darnell, | 4 00 |
| Jacob Lesher, | 2 00 |
| W. Smith, | 2 00 |
| H. Lathy, | 2 00 |
| M. Wooly, | 2 00 |
| W. J. LaRue, | 1 00 |
| H. C. Chapman, | 4 00 |
| J. N. Allen, | 4 00 |
| A. W. Heise, | 3 00 |
| Jas. E. Roberts, | 4 00 |
| S. Churchill, | 2 00 |
| L. S. Thompson, | 2 00 |
| Josiah Burrett, | 2 00 |
| Jno. B. Glass, | 4 00 |
| A. S. Hudson, | 4 00 |
| [illegible] Terwilliger, | 2 00 |
| J. J. H. Judd, | 2 00 |
| J Benett, | 2 00 |
| R. F. Adams, | 2 00 |
| H. Fisk, | 2 00 |
| J. M. Long, | 2 00 |
| B. G. Stevens, | 2 00 |
| S. N. Montague, | 2 00 |
| Dr. Hitchcock, | 2 00 |
| D. W. Boyd, | 4 00 |
| C. H. Quinlan, | 2 00 |
| **WISCONSIN.** | |
| Drs. Jno. Bartlett, | 6 00 |
| C. G. W. Bingham, | 2 00 |
| Clark & Rice, | 2 00 |
| J. Philips, | 1 50 |
| A. H. Vanorstrand, | 2 00 |
| J. H. Morrison, | 4 00 |
| C. A. Mills, | 2 00 |
| T. R. Kibbe | 2 00 |
| H. Gray, | 4 00 |
| B. Salisbury, | 3 00 |
| A. M. Warner, | 2 00 |
| H. W. Hart, | 2 00 |
| J. B. Waterbury, | 1 00 |
| M. B. Goff, | 4 00 |
| J. R. Bradway, | 2 00 |
| J. P. Hamilton, | 2 00 |
| Dr. Schoffield, | 2 00 |
| C. Pratt, | 2 00 |
| H. Loomis, | 4 00 |
| J. Prentice, | 3 00 |
| J. E. Thayer, | 5 00 |
| T. G. Cole, | 2 00 |
| W. Kimball, | 2 00 |
| G. Wright, | 2 00 |
| **MICHIGAN.** | |
| Drs. C. L. Henderson, | 2 00 |
| J. Smith, | 3 00 |
| N. M. Thomas, | 4 00 |
| L. B. Coates, | 2 00 |
| J. Snyder, | 2 00 |
| Dr. Peck, | 2 00 |
| J. C. Bardwell, | 4 00 |
| J. Wasson, | 2 00 |
| J. K. Finley, | 2 00 |
| A. Murray, | 2 00 |
| L. A. Brewer, | 2 00 |
| **OHIO.** | |
| J. S. Irvine, | 4 00 |
| C. W. Edwards, | 2 00 |
| A. H. Tyler, | 2 00 |
| J. N. Bentler, | 4 00 |
| **MARYLAND.** | |
| Dr. Thos. E. Bond, jr. | 2 50 |
| **IOWA.** | |
| Dr. S. G. Watson, | 4 00 |
| J. Dorer, | 2 00 |
| **NEW YORK.** | |
| Alb. Med. College, | 2 00 |
| Dr. Thos. Spencer, | 8 00 |

### TO SUBSCRIBERS.

With this number ceases the editorial connection with this Journal of Prof. Herrick, who has been so ong and favorably known to our readers. The Journal will be hereafter under the charge of the Editors whose names appear on the title page; to whose address should be sent all communications designed for either the editorial or publishing departments. Our thanks are due those of our subscribers who have remitted us the amount of our bills; but very many are yet in arrears: we hope they will reflect that although the individual amouuts are insignificant, the aggregate is a matter of much importance to us.

*Chicago, July 1st, 1849.*

